# Isolation, Characterization, and Multipotent Differentiation of Mesenchymal Stem Cells Derived from Meniscal Debris

**DOI:** 10.1155/2016/5093725

**Published:** 2016-12-04

**Authors:** Weili Fu, Xing Xie, Qi Li, Gang Chen, Chenghao Zhang, Xin Tang, Jian Li

**Affiliations:** ^1^Department of Orthopedics, West China Hospital, Sichuan University, Chengdu, China; ^2^Department of Orthopedic Surgery, Brigham and Women's Hospital, Harvard Medical School, Boston, MA, USA; ^3^Institute of Sports Medicine, Peking University Third Hospital, Beijing, China

## Abstract

This study aimed to culture and characterize mesenchymal stem cells derived from meniscal debris. Cells in meniscal debris from patients with meniscal injury were isolated by enzymatic digestion, cultured in vitro to the third passage, and analyzed by light microscopy to observe morphology and growth. Third-passage cultures were also analyzed for immunophenotype and ability to differentiate into osteogenic, adipogenic, and chondrogenic lineages. After 4-5 days in culture, cells showed a long fusiform shape and adhered to the plastic walls. After 10–12 days, cell clusters and colonies were observed. Third-passage cells showed uniform morphology and good proliferation. They expressed CD44, CD90, and CD105 but were negative for CD34 and CD45. Cultures induced to differentiate via osteogenesis became positive for Alizarin Red staining as well as alkaline phosphatase activity. Cultures induced to undergo adipogenesis were positive for Oil Red O staining. Cultures induced to undergo chondrogenesis were positive for staining with Toluidine Blue, Alcian Blue, and type II collagen immunohistochemistry, indicating cartilage-specific matrix. These results indicate that the cells we cultured from meniscal debris are mesenchymal stem cells capable of differentiating along three lineages. These stem cells may be valuable source for meniscal regeneration.

## 1. Introduction

The meniscus plays an important role, biochemically and biomechanically, in maintaining homeostasis of the knee [1]. Meniscus tears occur frequently as a result of aging or sports activity, and loss or damage of the meniscal structure can lead to knee degeneration [2]. However, the standard treatment for meniscal injury, partial or total meniscectomy, often leads to knee instability, degeneration, and dysfunction [3]. Alternatives are available but they come with several disadvantages. Suture repair is rarely used because of the limited blood supply in the remaining meniscal tissue and the relatively complex mechanical environment [4]. Meniscal allograft transplantation is considered the gold standard for treating patients with more serious meniscal defects and patients who have undergone meniscectomy, but long-term follow-up has shown limited and superficial host cell ingrowth. Transplantation also faces numerous challenges, including integrating the allograft with host tissue, obtaining limited donor tissue, preserving and processing donor tissue, achieving a match between donor and host meniscal size, fixing the grafted tissue, and avoiding infection and immune rejection [5]. The recently described procedure of meniscal xenogeneic transplantation shows promise for avoiding some of these problems, but much work is needed before it can be applied in the clinic [6].

An alternative to transplantation is meniscal regeneration through cell-based tissue engineering [7, 8]. The two main sources of cells for tissue engineering of meniscus are meniscal fibrocartilage cells and mesenchymal stem cells (MSCs) [9]. Autologous meniscal fibrocartilage cells derived from meniscectomized debris would in principle make the ideal seed cells for meniscal regeneration because the cells already have the proper cellular phenotype and they offer the potential for autologous therapy, with no additional risk of morbidity or immune rejection [10]. However, obtaining these cells requires two surgical procedures and the number of cells obtained is usually insufficient for seeding meniscal regeneration; in addition, the safety and efficacy of using meniscectomized debris in the clinic are unclear. Even more importantly, it is unclear whether these terminally differentiated mature fibrocartilage cells can function adequately in the long term in a rebuilt meniscus, since experiments in vitro have shown that their proliferative ability and biological activity gradually decrease with monolayer expansion and passaging, leading to loss of the phenotypic characteristics of primary meniscal fibrochondrocytes [11, 12].

Adult MSCs from mesoderm may prove even more effective for meniscal regeneration because they self-renew and show multilineage differentiation potential, immunomodulatory effects, and homing ability [13–15]. MSCs can differentiate into different mesenchymal or nonmesenchymal tissues under appropriate conditions in vitro and in vivo [16]. On the other hand, the cells do have disadvantages: they may undergo spontaneous transformation or senescence [17–20], and they may exhibit hypertrophy during chondrogenesis, resulting in apoptosis and ossification [21, 22]. Cartilage formed by MSCs has inferior content in extracellular matrix and does not provide the same mechanical properties as cartilage formed by mature chondrocytes [23]. In addition, MSCs account for only 0.001–0.01% of mononuclear cells in bone marrow [24]. It would therefore be useful to identify alternative sources of MSCs for meniscal repair and reconstruction.

MSCs are found in nearly all tissues of the body, not just in bone marrow [25]. For example, MSCs have been isolated from adipose tissue and synovium for meniscal regeneration [26, 27], but harvesting cells from these sources is associated with donor site morbidity. More importantly, these cells give unsatisfactory outcomes for meniscal regeneration because the regulatory pathways controlling their differentiation are poorly understood. Studies suggest that the injured meniscus itself may show certain healing potential [28], so we speculate that MSCs exist within the meniscal debris. These cells may offer the combined advantages of both meniscal fibrocartilage cells and mesenchymal cells.

To explore whether meniscal debris is a good source of seed cells for meniscal regeneration, we used enzymatic digestion to isolate cells in meniscal debris from patients with meniscal injury, and then we identified these cells as MSCs based on their adherent ability, morphology, phenotype, and multilineage differentiation potential. Such cells may provide adequate amounts of seed cells for meniscal regeneration.

## 2. Materials and Methods

### 2.1. Isolation and Culture of MSCs from Meniscal Debris

This study was approved by the local Research Ethics Committee, and written informed consent was obtained from all patients. Meniscal debris was collected from 6 patients with meniscal tears (3 men and 3 women; 6 knees, comprising 4 left and 2 right), who underwent arthroscopic partial meniscectomy or plasty. They were diagnosed on the basis of clinical manifestations, magnetic resonance imaging, and arthroscopy. Their median age was 37 years (25–49). The causes of meniscus tears were related to either sport activities (5 patients) or degeneration (1 patient). There were 5 lateral and 1 medial menisci. The median time of surgery after injury was 22 months (2–60). The tears included horizontal (2 menisci), longitudinal (2 menisci, including 1 bucket-handle tear), flap (1 meniscus), and complex (1 meniscus horizontal + longitudinal tears) tears. The tear areas were located either in the anterior horn (2 menisci) or in the meniscal body (4 menisci). The tears involved the white zone (4) or red-white (2) zone. Two patients had concomitant cartilage lesions.

Meniscal debris-derived MSCs were isolated and cultured as previously described [29]. Briefly, meniscal debris was rinsed with phosphate-buffered saline (PBS) containing 1% gentamicin to remove surrounding tissue, then minced into 0.5-mm^3^ pieces, and digested with 2 mg/mL 1 : 1 mixed collagenase type I and type II for 4–6 h at 37°C. The isolated cells were washed three times with PBS and suspended to a concentration of 1 × 10^6^ cells/mL in complete low-glucose Dulbecco's modified Eagle's medium (LG-DMEM) containing 10% fetal bovine serum (FBS), 2.2 g NaHCO_3_, 100 U/mL penicillin, 100 *μ*g/mL streptomycin, 25 ng/mL amphotericin B, and 2 mM l-glutamine at 37°C in 5% humidified CO_2_. After 48–72 h, nonadherent cells were washed away with PBS. The medium was replaced every 3-4 days. When the primary cultures reached 80–90% confluence, they were trypsinized with 0.25% trypsin/0.1% EDTA and passaged by splitting with the ratio of 1 : 2 or 1 : 3. Third-passage (P3) cultures were utilized for subsequent experiments.

### 2.2. Phenotypic Characteristics of MSCs from Meniscal Debris

Potential markers expressed on the surface of third-passage MSCs derived from meniscal debris were analyzed by flow cytometry. Cells were harvested with trypsin/EDTA, then were incubated for 1 h with FITC-conjugated antibodies against CD44 (Abcam), or purified primary antibodies against CD90, CD105, CD34, or CD45 (Abcam). Next, cells were labeled for 30 min with FITC-conjugated secondary antibody. In parallel, cells were incubated with nonspecific mouse IgG instead of primary antibody to detect nonspecific staining. Then cells were fixed in flow buffer, washed, and subjected to flow cytometry and results were analyzed using Cell Quest software (BD Biosciences). The results were expressed as percentages of positive cells on histogram plots relative to the proportions obtained with the isotype-matched negative control.

### 2.3. Trilineage Differentiation Potential of MSCs Derived from Meniscal Debris

The potential of third-passage MSCs from meniscal debris to differentiate along three lineages was examined, as previously described [30].

#### 2.3.1. Osteogenesis

MSCs were grown to 80–90% confluence and then induced for 2 weeks in osteogenic medium supplemented with 0.1 *μ*M dexamethasone, 10 mM *β*-glycerol phosphate, and 50 *μ*M ascorbate. Cultures were considered positive for osteogenesis if they showed alkaline phosphatase (ALP) activity and the presence of Alizarin Red-positive calcium deposits.

#### 2.3.2. Adipogenesis

MSCs were induced for 2 weeks in adipogenic medium consisting of 1 *μ*M dexamethasone, 0.5 mM methyl-isobutylxanthine, 10 g/mL insulin, and 100 mM indomethacin. Cultures were considered positive for adipogenesis cells if they showed the accumulation of Oil Red O-stained lipid vacuoles within the cytoplasm.

#### 2.3.3. Chondrogenesis

MSCs (10^6^ cells) were collected in 15-mL polypropylene centrifuge tubes, centrifuged at 480 ×g for 10 min, and cultured in micromass for 3 weeks at 37°C with 5% CO_2_ in high-glucose DMEM supplemented with 100x ITS, 1 mmol/L pyruvate, 0.17 mmol/L ascorbate, 0.1 *μ*M dexamethasone, 0.35 mmol/L proline, and 10 ng/mL TGF*β*3. Sagittal sections were processed with hematoxylin and eosin to reveal general histology, Toluidine Blue and Alcian Blue to detect glycosaminoglycan (GAG), and immunohistochemistry to detect expression of type II collagen (Col-II).

### 2.4. Real-Time Quantitative PCR

After inducing MSCs cultures to follow one of the three lineages described above, they were assayed for expression of specific markers for extracellular matrix and transcription factors using real-time quantitative PCR. Total RNA from samples was extracted using RNAVzol reagent (Vigrous) according to the manufacturer's instructions. Concentration of each RNA sample was measured by UV spectrophotometry and integrity of RNA samples was assessed by agarose gel electrophoresis. Total RNA (2 *μ*g) was subjected to reverse transcription using the Superscript First Strand Synthesis System (Invitrogen). PCR primers were designed using Oligo6 primer analysis software (Table 1). Quantitative real-time PCR was performed in 15-*μ*L reactions consisting of 7.5 *μ*L 2x SYBR Green PCR Master Mix (Toyobo), 1 *μ*L cDNA product, 1 *μ*L of each primer, and 5.5 *μ*L of nuclease-free water. All PCRs were performed under the following conditions: 2 min at 50°C, 10 min at 95°C, and 40 cycles of 15 s at 95°C and 1 min at 60°C. ABI Prism 7000 Sequence Detection System software was used to perform melting curve analysis to verify amplification specificity and determine mRNA levels using the comparative cycle threshold (Ct) method. Expression levels of mRNA were normalized to that of GAPDH mRNA using the same Ct method.

### 2.5. Statistical Analysis

Expression levels of each mRNA were reported as mean ± SD (*n* = 6). Student's* t*-test was used to assess the significance of differences between induced cultures on a given day and uninduced controls on day 0. *P* < .05 was considered statistically significant.

## 3. Results

### 3.1. Isolation and Culture of MSCs Derived from Meniscal Debris

**Figure 1:**
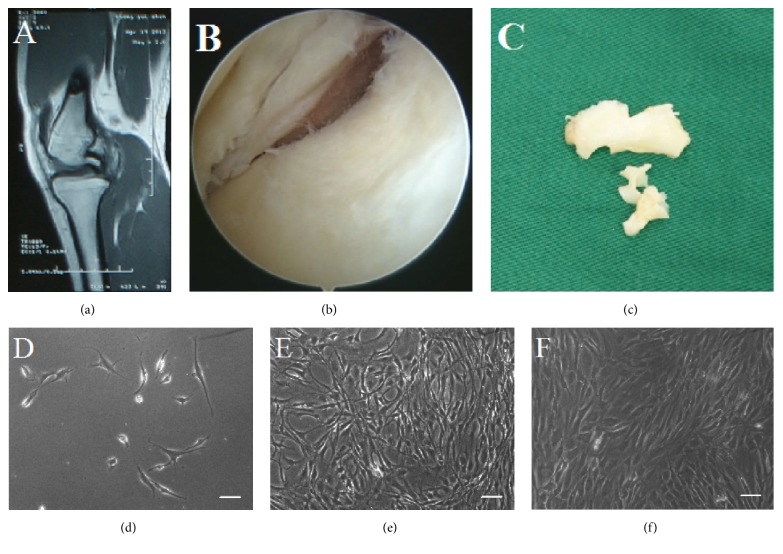
Magnetic resonance imaging (a) showing a typical bucket-handle tear of medial meniscus in sagittal view and arthroscopic examination (b) of patients with meniscal tears. Meniscal debris (c) was obtained during surgery. At 3~5 d after plating, some cells were observed to adhere to the culture dish (d). Cells took on a swirling or cluster appearance by 10–12 d (e), and they remained homogeneous until the third passage (f). Scale bar = 100 *μ*m.

Nucleated cells were isolated from the meniscal debris using collagenase. After primary cultures had been in the incubator for 2-3 d, the isolated round cells gradually spread out and adhered to the culture dish. By 4-5 d, cells exhibited typical spindle-shaped, fibroblast-like morphology. As time went on, the cells grew more rapidly and took on a swirling or cluster appearance. By 10–12 d, cultures reached 80–90% confluence, whereupon they were subcultured 1 : 2 or 1 : 3. Following the first subculturing, approximately 4-5 days were needed for each passage. Cell morphology remained homogeneous until P3 (Figure 1).

### 3.2. Phenotypic Characteristics of MSCs Derived from Meniscal Debris

**Figure 2:**
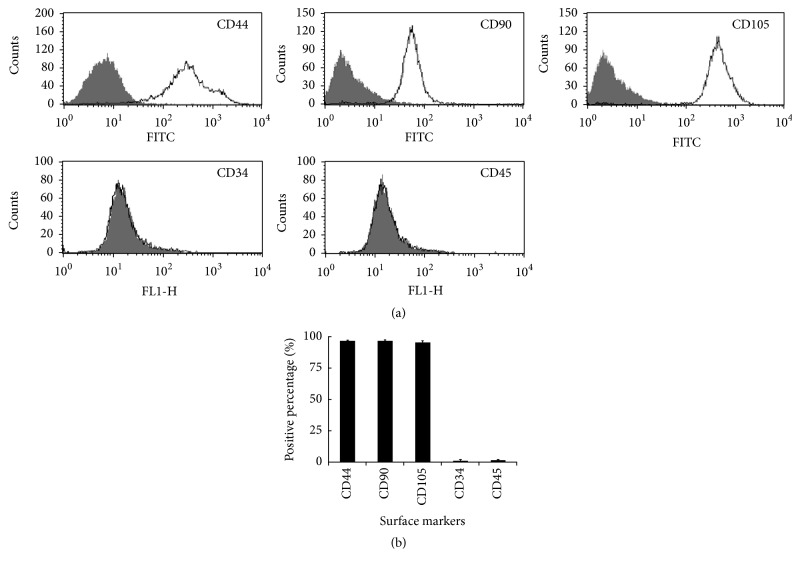
Phenotypic characteristics of MSCs derived from meniscal debris, based on flow cytometry. Cells were labeled with antibodies against CD44, CD90, CD105, CD34, and CD45. The black lines correspond to fluorescence intensity of anti-marker antibodies, while grey areas correspond to nonspecific anti-mouse IgG as an isotype control (a). Data are presented as mean ± SD (b).

Flow cytometry indicated that nearly all third-passage MSCs derived from meniscal debris were positive for the surface markers CD44 (96.27 ± 2.73%), CD90 (96.46 ± 1.86%), and CD105 (95.4 ± 3.23%). At the same time, the cells showed minimal surface expression of CD34 (0.99 ± 1.07%) and CD45 (1.3 ± 0.81%) (Figure 2).

### 3.3. Trilineage Differentiation Potential of MSCs Derived from Meniscal Debris

**Figure 3:**
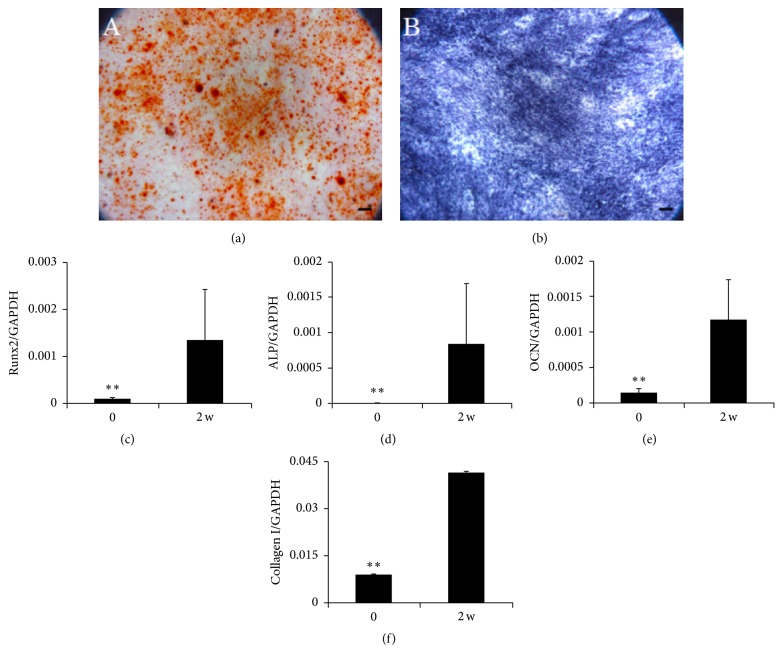
Osteogenic differentiation potential of third-passage MSCs cultures derived from meniscal debris. After 14-day induction in osteogenic medium, osteogenesis was determined based on deposition of matrix calcification detected by Alizarin Red (a) and based on ALP-specific staining (b). Scale bar = 100 *μ*m. Expression of osteogenic genes encoding Runx2 (c), ALP (d), OCN, (e) and type I collagen (f) was assessed using real-time PCR. Data are presented as mean ± SD. ^*∗∗*^ *P* < .01.

Third-passage MSC cultures were subjected to in vitro differentiation assays in order to investigate their mesenchymal multipotency potential. When cultures were induced to undergo osteogenesis, the cells began to gather and become sparse, changing from a spindle-shaped morphology to a more polygonal one. After 1-2 weeks, the nodules became larger and scattered uniformly. After 14 d, cultures were stained with Alizarin Red (Figure 3(a)) to detect calcification and stained for ALP (Figure 3(b)) to detect the presence of osteoblasts. These tests showed that MSCs secreted bone matrices and the osteoblast marker ALP. Bone matrices were stained red in the presence of Alizarin Red. Cultures also showed scattered, uniformly distributed mineral nodules. Quantitative PCR analysis further showed that expression levels of osteogenic-specific genes for Runx2, ALP, OCN, and Col-I were significantly higher in induced cultures than in uninduced ones (Figures 3(c)–3(f)).

**Figure 4:**
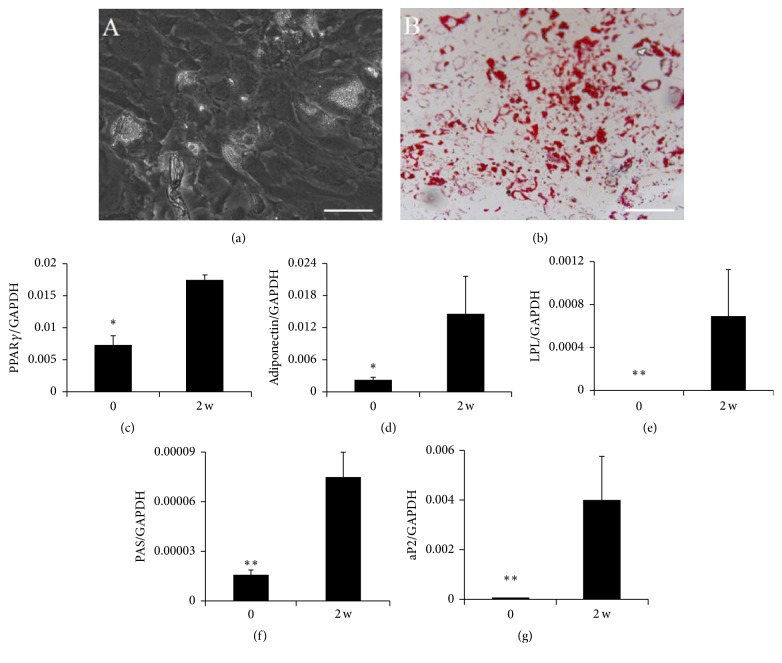
Adipogenic differentiation potential of third-passage MSCs cultures derived from meniscal debris. After 14-day induction in adipogenic medium, adipogenesis was detected as the formation of neutral lipid vacuoles (a) stainable with Oil Red O (b). Scale bar = 200 *μ*m. Expression of adipogenic genes PPAR*γ* (c), adiponectin (d), LPL (e), PAS (f), and aP2 (g) was assessed by real-time PCR. Data are presented as mean ± SD. ^*∗*^ *P* < .05; ^*∗∗*^ *P* < .01.

When third-passage cultures were induced to undergo adipogenesis, the volumes of cells and nuclei increased, and intracellular lipid droplets became visible in the cytoplasm by microscopy (Figure 4(a)). These droplets were confirmed to be lipid vacuoles because they stained red with Oil Red O (Figure 4(b)). Lipids continued to accumulate during 2 weeks. Quantitative PCR analysis further showed that expression levels of adipogenic-specific genes for PPAR-*γ*, Adiponectin, LPL, PAS, and aP2 were significantly higher in induced cultures than in uninduced ones (Figures 4(c)–4(g)).

**Figure 5:**
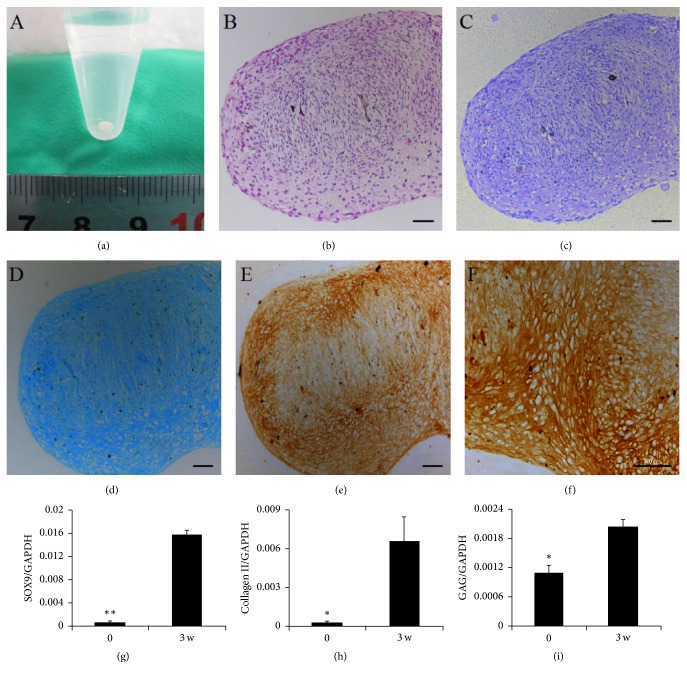
Chondrogenic differentiation potential of third-passage MSCs cultures derived from meniscal debris. At 21 day after chondrogenic induction, chondrogenesis was induced under serum-free micromass pellet culture conditions (a) and analyzed using hematoxylin and eosin staining (b) for general histology, Toluidine Blue (c) and Alcian Blue staining (d) for GAG, and immunohistochemical staining for type II collagen in chondrogenic matrix ((e): low-magnification; (f): high-magnification). Scale bar = 100 *μ*m. Expression of chondrogenic genes SOX9 (g), type II collagen (h), and GAG (i) was assessed by real-time PCR. Data are presented as mean ± SD. ^*∗*^ *P* < .05; ^*∗∗*^ *P* < .01.

When third-passage cultures were induced to undergo chondrogenesis, pellets changed to spheroids (Figure 5(a)). Hematoxylin and eosin staining (Figure 5(b)) showed that cells were round like chondrocytes with lacuna-like structures. The micromasses became rich in GAG and Col-II, based on strong staining with Toluidine Blue (Figure 5(c)), Alcian Blue (Figure 5(d)), and immunohistochemistry (Figures 5(e) and 5(f)). Staining with Toluidine Blue and Alcian Blue also revealed a considerable degree of metachromasia. Quantitative PCR analysis further showed that expression levels of chondrogenic-specific genes for SOX-9, Col-II, and GAG were significantly higher in induced cultures than in uninduced ones (Figures 5(g)–5(i)).

## 4. Discussion

In this study, we successfully isolated adherent cells from meniscal tear debris; the cells had a morphology typical of fibroblasts and they expressed a characteristic mesenchymal phenotype, with no expression of hematopoietic surface markers. It was possible to effectively induce the cultured cells to differentiate into osteogenic, adipogenic, or chondrogenic lineages. The cells were identified as MSCs based on their morphology, surface marker expression, ability to adhere to culture surfaces, and multilineage differentiation potential. Our findings suggest that meniscal debris may be a useful source of seeding cells for meniscal regeneration.

Some studies showed that cells in the vascular periphery of meniscal injury, or even in the avascular area, could spontaneously heal the tissue damage [31]. This suggested that meniscal debris may contain stem or progenitor cells that can participate in meniscal regeneration. These cells may come from several sources. One possible source is from a nearby area such as synovial fluid: MSCs increase in greater numbers in synovial fluid after meniscus injury than in normal knees even within the avascular area [32]. Another possible source of MSCs is the meniscus itself. Following injury or certain pathological conditions, MSCs may be activated and recruited to injured tissues, where they assist in homeostasis, remodeling, and repair by replacing mature cells that have been lost and by exerting paracrine effects to recruit cells to the site of injury [33]. MSCs populations may have multiple origins (systemic or local origin), as suggested by studies in which MSCs were shown to arise from diverse mesenchymal lineages [34, 35]. Another basis of MSCs existence in meniscus tissue may be that MSCs could be used to investigate the pathogenesis of meniscal calcification or ossicle [36, 37].

Whatever the origin of the MSCs in our meniscal debris samples, their properties are similar to those previously reported for human meniscus stem/progenitor cells, which displayed characteristics of MSCs and expressed high levels of Col-II [38, 39]. Those cells promoted meniscus regeneration and ameliorated osteoarthritis through SDF-1/CXCR4-mediated homing in a rat model of meniscus injury. How meniscal tissue-specific MSCs are activated to regenerate meniscal tissue and what mechanisms they use during that regeneration are still unclear. Whatever the pathways involved, our results suggest that meniscal tissue-specific MSCs may be superior to terminally differentiated mature cells and to MSCs derived from other sources for meniscal regeneration.

## 5. Conclusions

We have isolated cells from human meniscal debris that were fibroblast-like and that were able to adhere to plastic and undergo several passages in vitro. The cells showed a distribution of surface markers similar to that previously reported for MSCs. They were also efficiently induced to differentiate into osteoblasts that produced mineralized matrix, adipocytes that accumulated lipid vacuoles, and chondrocytes that produced GAG and Col-II. Real-time PCR analysis confirmed that each differentiated lineage upregulated the corresponding genes for osteogenesis, adipogenesis, or chondrogenesis. Our study demonstrated the existence of MSCs in meniscal debris and showed that they can be cultured and differentiated, opening the door to studies examining their potential for meniscal regeneration.

## Figures and Tables

**Table 1:** Primer sequences for quantitative real-time PCR.

Target gene	Primer sequence (5′-3′)	
	Forward	Reverse
Runx2	TGGTTACTGTCATGGCGGGTA	TCTCAGATCGTTGAACCTTGCTA
ALP	ACCACCACGAGAGTGAACCA	CGTTGTCTGAGTACCAGTCCC
OCN	GGCGCTACCTGTATCAATGG	GTGGTCAGCCAACTCGTCA
Collagen I	GAGGGCCAAGACGAAGACATC	CAGATCACGTCATCGCACAAC
PPAR*γ*	GATGCCAGCGACTTTGACTC	ACCCACGTCATCTTCAGGGA
Adiponectin	GGTGCTGAAGCCTACCAAC	AGGAAGAACAGACGGCAGAAC
LPL	TCATTCCCGGAGTAGCAGAGT	GGCCACAAGTTTTGGCACC
PAS	AAGGACCTGTCTAGGTTTGATGC	TGGCTTCATAGGTGACTTCCA
aP2	ACTGGGCCAGGAATTTGACG	CTCGTGGAAGTGACGCCTT
SOX9	AGCGAACGCACATCAAGAC	CTGTAGGCGATCTGTTGGGG
Collagen II	TGGACGCCATGAAGGTTTTCT	TGGGAGCCAGATTGTCATCTC
GAG	ACTCTGGGTTTTCGTGACTCT	ACACTCAGCGAGTTGTCATGG
GAPDH	ACACTCAGCGAGTTGTCATGG	ACACCATGTATTCCGGGTCAAT
